# KLRF1, a novel marker of CD56^bright^ NK cells, predicts improved survival for patients with locally advanced bladder cancer

**DOI:** 10.1002/cam4.5579

**Published:** 2022-12-29

**Authors:** Neelam Mukherjee, Niannian Ji, Xi Tan, Chun‐Liang Chen, Onika D. V. Noel, Maria Rodriguez‐Padron, Chun‐Lin Lin, David G. Alonzo, Tim H. Huang, Robert S. Svatek

**Affiliations:** ^1^ Department of Urology University of Texas Health San Antonio (UTHSA) San Antonio Texas United States; ^2^ Department of Molecular Medicine University of Texas Health San Antonio (UTHSA) San Antonio Texas United States; ^3^ Department of Urology University of Texas Rio Grande Valley (UTRGV) Edinburg Texas United States

**Keywords:** bladder cancer, CD56, KLRF1, NK cells, survival

## Abstract

**Background:**

Bladder tumor‐infiltrating CD56^bright^ NK cells are more tumor cytotoxic than their CD56^dim^ counterparts. Identification of NK cell subsets is labor‐intensive and has limited utility in the clinical setting. Here, we sought to identify a surrogate marker of bladder CD56^bright^ NK cells and to test its prognostic significance.

**Methods:**

CD56^bright^ and CD56^dim^ NK cells were characterized with the multiparametric flow *(n = 20)* and mass cytometry (*n* = 21) in human bladder tumors. Transcriptome data from bladder tumors (*n* = 351) profiled by The Cancer Genome Atlas (TCGA) were analyzed. The expression levels of individual markers in intratumoral CD56^bright^ and CD56^dim^ NK cells were visualized in tSNE plots. Expressions of activation markers were also compared between Killer Cell Lectin‐Like Receptor Subfamily F Member 1 (KLRF1)^+^ and KLRF1^−^ NK cells.

**Results:**

Intratumoral CD56^bright^ NK cells displayed a more activated phenotype compared to the CD56^dim^ subset. Multiple intratumoral cell types expressed CD56, including bladder tumor cells and nonspecific intratumoral *CD56* expression was associated with worse patient survival. Thus, an alternative to CD56 as a marker of CD56^bright^ NK cells was sought. The activation receptor KLRF1 was significantly increased on CD56^bright^ but not on CD56^dim^ NK cells. Intratumoral KLRF1^+^ NK cells were more activated and expressed higher levels of activation molecules compared with KLRF1^−^ NK cells, analogous to the distinct effector function of NK cells across CD56 expression. High intratumoral *KLRF1* was associated with improved recurrence‐free survival (hazard ratio [HR] 0.53, *p* = 0.01), cancer‐specific survival (HR 0.47, *p* = 0.02), and overall survival (HR 0.54, *p* = 0.02) on multivariable analyses that adjusted for clinical and pathologic variables.

**Conclusions:**

KLRF1 is a promising prognostic marker in bladder cancer and may guide treatment decisions upon validation.

## Graphical abstract

Intratumoral CD56^bright^ NK cells displayed a more activated phenotype compared to the CD56^dim^ subset in bladder cancer. KLRF1 is a surrogate marker of CD56^bright^ NK cells and is a potential prognostic marker in bladder cancer.

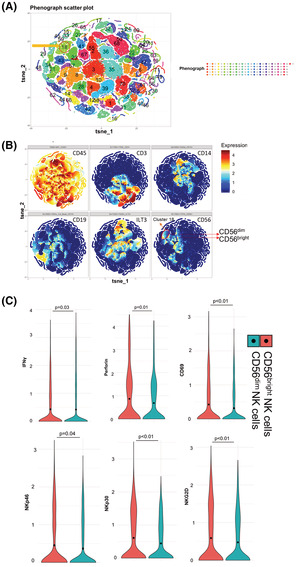

## 1 INTRODUCTION

The immune system plays a critical role in preventing the development and progression of many solid tumors, including urinary bladder cancer. Antitumor immune mechanisms are numerous and include contributions from both the innate and adaptive immune systems. Natural killer (NK) cells recognize and kill stressed cells, including virus‐infected and cancer cells.^1^ NK cells are classically described as innate immune cells due to their capacity to lyse target cells without the need for prior antigen sensitization.^2^ Although NK cells represent a small percentage of total intratumoral lymphocytes, their presence is associated with improved survival in several solid malignancies.^3^, ^4^, ^5^

We previously identified a favorable prognostic significance of NK cells expressing high amounts of CD56 within tumors from patients with invasive bladder cancer.^6^ The majority of bladder intratumoral NK cells were CD56^dim^ and the proportion of CD56^dim^ NK cells increased in higher stage tumors. Intratumoral CD56^dim^ NK cells were less cytotoxic than intratumoral CD56^bright^ NK cells and the presence of CD56^bright^ NK cells in bladder tumors was independently associated with improved patient survival. These findings support CD56^bright^ NK cells as a novel prognostic biomarker for patients with bladder cancer. However, identification of these NK cell subsets with multiparametric flow cytometry is labor‐intensive and has limited utility in the clinical setting. Here, we sought to identify a surrogate marker of bladder tumor‐infiltrating CD56^bright^ NK cells that could be used for tumor prognostication.

NK cells tightly regulate their cytotoxic capacity by integrating environmental signals through activating and inhibitory molecules, including immunoglobulin‐like or C‐type lectin‐like receptors, which bind corresponding activating and inhibitory ligands on target cells.^7^ Here, multiparametric flow and mass cytometry was used to identify a novel surrogate marker of activated CD56^bright^ NK cells and characterize intratumoral lymphocytes isolated from bladder tumors in patients with muscle‐invasive bladder cancer undergoing radical cystectomy.

## 2 MATERIALS AND METHODS

### 2.1 Bladder cancer patient cohort

Patients were recruited through a local Institutional Review Board (IRB)‐approved observational cohort study, which prospectively collected clinical data and bladder tissue for analysis (IRB # BCR20120159H). Eligible patients were 18 years of age or older and had a confirmed or suspected diagnosis of bladder cancer. All patients provided written informed consent. Patient demographics, pathology and imaging reports, physical exam and laboratory assessments, and specimen tracking data were entered prospectively into a secured web‐based REDCap database system. This study's involvement with human subjects complies with the Declaration of Helsinki.

### 2.2 Human bladder tumor sample processing

As described,^8^ bladder tumors were surgically excised under sterile conditions as per standard of care. A portion of the tumor was separated and placed in Roswell Park Memorial Institute (RPMI) 1640 medium containing 1% antibiotic (Penicillin–Streptomycin) and transported on ice. Fresh tumor tissues were washed with phosphate‐buffered saline (PBS) and minced into 1–2 mm pieces and incubated in digestion solution (1 mg/mL collagenase, 0.25% trypsin, and 0.25 mg/mL DNAse) for 40 min at 37°C, 5% CO_2_. After digestion, the enzymes are neutralized by the addition of complete RPMI containing 10% fetal bovine serum (FBS), and the samples were filtered through a 100 μM filter to produce single‐cell suspensions. Single‐cell suspensions were cryopreserved and stored at −150°C until analyzed.

### 2.3 Cytometry by time of flight (CyTOF) staining

CyTOF staining was conducted using single‐cell suspensions derived from bladder tumor specimens (*n* = 21 patients with muscle‐invasive [≥T2] urothelial carcinoma of the bladder, Table 1) according to manufacturer's instructions. To define the phenotypic diversity of human bladder tumor innate lymphoid cells, we designed a CyTOF panel of 36 antibodies **(**Table S1).^8^ As described,^8^ cells were thawed in Hank's balanced salt solution without Ca2^+^ or Mg2^+^ + 10% FBS and the number of viable cells was quantified using trypan blue. Before surface staining, cells were stained with cisplatin or discrimination of dead cells from live cells. Cells were then stained first with the surface antibody cocktail for 30 min (see Table S1 for clone list and metal). After washing, cells were fixed and permeabilized with MaxPerm‐S buffer for 30 min before staining with the intracellular antibody cocktail for 30 min. After washing steps, cells were stained for Cell‐ID Intercalator‐Ir to discriminate single nucleated cells from doublets. Finally, cells were resuspended in Cell Acquisition Solution (CAS)‐bead solution to 1 million cells/mL before the acquisition of data on Helios. Purified antibodies lacking carrier proteins were conjugated using the Maxpar labeling kit and according to the protocol provided by Fluidigm.

**TABLE 1:** Characteristics of patient cohorts. The clinical parameters of the selected bladder patient cohorts used for CyTOF staining (*n* = 21), flow staining (*n* = 20), and TCGA analysis (*n* = 351) are listed

Variables	Cohort *n* = 21	Cohort *n* = 20	Cohort *n* = 351
Median age (IQR)	76 (63.5–80)	72 (63–81.5)	69 (60–77)
Gender			
Female	4 (19.05%)	7 (35%)	94 (26.8%)
Male	17 (80.95%)	13 (65%)	257 (73.2%)
Stage			
T0			1 (0.28%)
T1			3 (0.85%)
T2	12 (57.14%)	6 (30%)	107 (30.48%)
T3	6 (28.57%)	8 (40%)	183 (52.14%)
T4	3 (14.29%)	6 (30%)	57 (16.24%)
Histologic subtype			
Pure urothelial carcinoma	13 (61.90%)	12 (60%)	
Urothelial carcinoma with squamous differentiation	7 (33.33%)	4 (20%)	
Urothelial carcinoma with small cell carcinoma	1 (4.76%)	2 (10%)	
Urothelial carcinoma with plasmacytoid differentiation		1 (5%)	
Urothelial carcinoma with sarcomatoid differentiation		1 (5%)	
Luminal			185 (52.71%)
Nonluminal			166 (47.29%)
Prior chemotherapy			
Yes	8 (38.10%)	5 (25%)	62 (17.7%)
No	13 (61.90%)	15 (75%)	159 (45.3%)
Unknown			130 (37.0%)
Prior radiation			
Yes	3 (14.29%)	2 (10%)	230 (65.5%)
No	18 (85.71%)	18 (90%)	6 (1.7%)
Unknown			115 (32.8%)

### 2.4 CyTOF data analysis

CyTOF data in FCS format were first processed in Cytobank (Cytobank, Inc) to gate CD45^+^ cells. The CD45^+^ cells were further analyzed in an R package Cytofkit for clustering and visualization.^9^ The subclusters of the cells were decided by a graph‐based partitioning method. PhenoGraph in the package. The NK cells were then identified based on the intensity of selected markers (CD45^+^, CD3^−^, CD14^−^, CD19^−^, ILT3^−^, and CD56^bright/dim^) as described.^6^, ^8^ The expression levels of individual markers were visualized in tSNE plots. The comparison of expression levels of individual markers was displayed in the violin plot using the *ggplot* function in R.

### 2.5 Flow staining

Intratumoral cells were stained (see Table S2 for clone list and fluorochromes) and analyzed^6^, ^10^ using Cytek® full spectrum flow cytometry systems and single‐cell suspensions derived from bladder tumor specimens (*n* = 20 patients, Table 1). Single‐cell suspensions were mixed with Brefeldin A as a Golgi blocker for 5 h before cytokine staining. Compensation matrices were calculated automatically, and sample analysis was carried out using FlowJo software. Antibodies and dyes are tabulated in Table S2. Mean fluorescent intensity (MFI) was used to compare the expression levels of specific genes across different cell populations.

### 2.6 The cancer genome atlas (TCGA) patient dataset

Transcription levels as RPKM (reads per kilobase of transcript per million mapped reads) of subtype signature genes (*n* = 45) of bladder tumors from patients were downloaded from the TCGA data portal and normalized as Z scores.^11^, ^12^ From the *initial patient cohort of n* = 408, a total of 57 were excluded due to missing data regarding gene expression, pathologic stage, or follow‐up time, leaving *n* = 351 *patients* for the final analyses (for clinicopathologic characteristics, see Table 1).

### 2.7 Killer cell lectin‐like receptor subfamily F member 1 (KLRF1) evaluation in bladder cancer patient cohort (TCGA)

The markers for NK cells and MHC I in bladder cancer cells were followed with modifications as derived from a pan‐cancer meta‐analysis previously.^13^ Z scores were converted into a positive scale for comparison convenience. NK cells were identified in TCGA dataset by the expression of *KLRF1*. The gene expression and clinical data of another patient cohort^14^ were downloaded from CBioPortal.^15^

### 2.8 Statistical analysis

The proportion of intratumoral NK cells between any two groups was compared using Mann–Whitney tests or unpaired *t*‐tests. The Kaplan–Meier method was used to graph survival across NK cell subsets. The log‐rank (Mantel‐Cox) test compared survival distributions between groups. The *p*‐values in the comparison of expression levels of individual markers (violin plots) between CD56^bright^ and CD56^dim^ NK cells were calculated by the Wilcoxon rank sum test.

Single variable and multivariable Cox proportional hazards regression models were used to identify associations with recurrence‐free survival (RFS), cancer‐specific survival (CSS), and overall survival (OS). A clinical and demographic Cox model for survival was built by including all such variables (i.e., age, gender, pathologic stage, and tumor subtype) that had significant associations with survival and then fitted with the variable NK cells. Associations with survival outcomes were examined using both continuous and discreet variables for NK cells defined by the population median and by two quantiles, splitting the population into three groups. Grouping NK cells into tertiles (i.e., low, mid, and high) provided the most parsimonious model.

To evaluate for effect modification, we stratified analyses by tumor MHC I expression and tested for heterogeneity by fitting the model with an interaction term for NK cells and MHC I (NK cells‐MHC I). A multivariable model including adjuvant therapy (*n* = 221) included fewer patients due to missing data regarding receipt of adjuvant therapy and are shown in the Table S4. In all models, proportional hazards assumptions were systematically verified using the Grambsch–Therneau residual‐based test. For all analyses, a *p* ≤ 0.05 was considered statistically significant and all *p*‐values were two‐sided. Statistical analyses were performed using Stata/IC 10.1, GraphPad Prism 6, or R 3.3.0.

## 3 RESULTS

**FIGURE 1:**
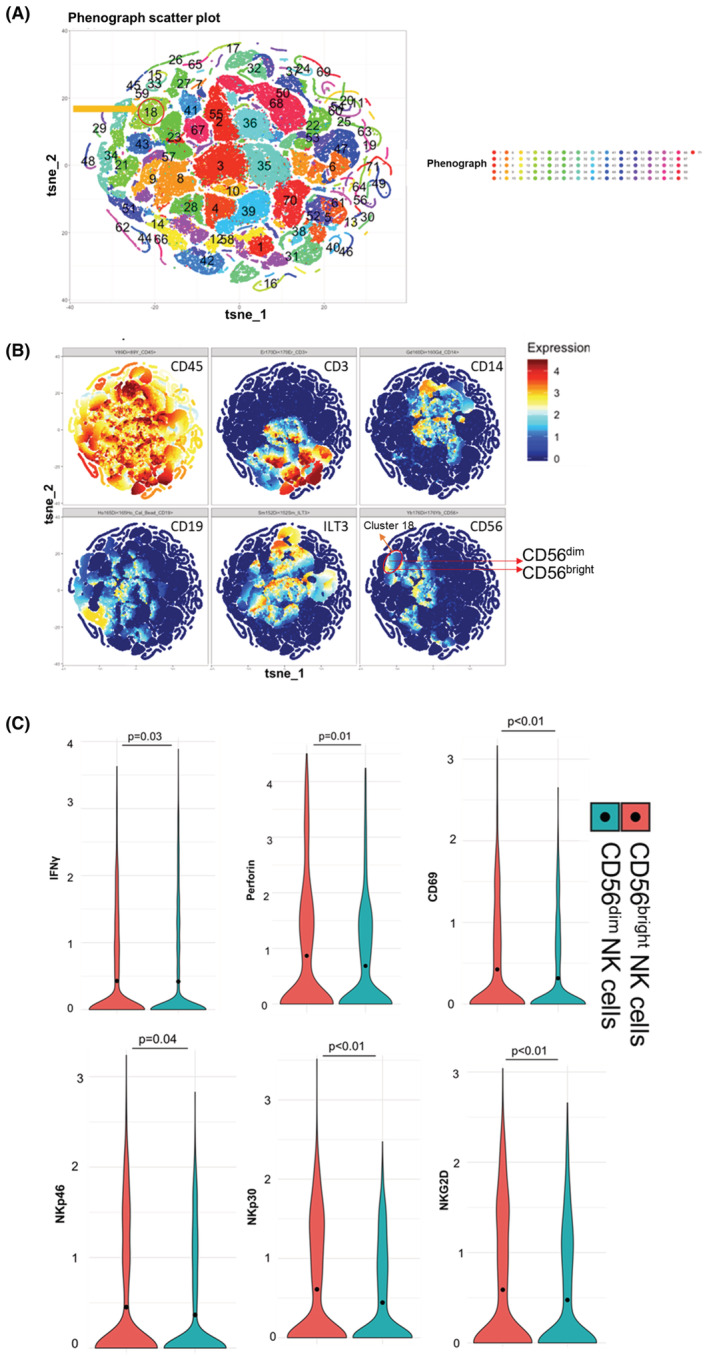
Bladder tumor‐infiltrating CD56^bright^ NK cells expressed a significantly higher level of activation molecules compared with CD56^dim^ NK cells. Human bladder tumor tissues (*n* = 21) were harvested and processed into single‐cell suspensions and analyzed with CyTOF. (A) The CD45^+^ cells were analyzed in an R package Cytofkit for clustering and visualization [11]. The subclusters of the cells were decided by a graph‐based partitioning method, PhenoGraph in the package. (B) The NK cells were then identified based on the intensity of selected markers (CD45^+^, CD3^−^, CD14^−^, CD19^−^, ILT3^−^, and CD56^dim^/^bright^). The expression levels of individual markers were visualized in tSNE plots. The t‐SNE plots showing the expression of different markers are used in the identification of NK cells. (C) Different expressions of cytokines and activation receptors in CD56^bright^ and CD56^dim^ NK cells are plotted as violin plots. Wilcoxon rank sum test. Functions of each gene: IFNγ is involved in increasing the activation and cytotoxicity of NK cells; NKp30, NKp46, and NKG2D are activation receptors involved in the functional activation of NK cells; early activation marker CD69 activates NK cells and triggers NK cell‐mediated cytotoxicity; cytotoxic molecule perforin helps in the formation of pores in membranes of target cells at the immunologic synapse between NK cells and target cells and facilitates NK cell‐mediated cytolytic activity.

CD56^bright^ and CD56^dim^ intratumoral NK cell subsets in bladder tumors were identified by polychromatic mass cytometry based on their CD56 expression and the absence of lineage markers within the CD45^+^ lymphocyte gate. Unbiased clustering of human bladder lymphocytes revealed a distinct cluster of CD45^+^CD14^−^CD19^−^CD3^−^ILT3^−^ NK cells (cluster 18; Figure 1A, B), visualized using a *t*‐distributed stochastic neighbor embedding (t‐SNE) plot where each dot represents a single cell. As expected, cluster 18, comprising NK cells, showed a high expression of CD45 and no expression of non‐NK lineage markers (Figure 1B). Further, we identified two primary NK cell subsets: CD56^bright^ and CD56^dim^ NK cells in cluster 18 (Figure 1B), and characterized the two subsets using functional markers including antitumor and inflammatory cytokines and markers of activation and cytotoxicity. CD56^bright^ NK cells displayed a more activated phenotype compared to the CD56^dim^ subset, characterized by increased expression of IFNγ (*p* = 0.03), activation receptors (NKp30, NKp46, NKG2D; *p* < 0.05), early activation marker CD69 (*p* < 0.01), and cytotoxic molecule perforin (*p* = 0.01) (Figure 1C). These findings support CD56^bright^ NK cells in bladder tumors as functionally more active than CD56^dim^ NK cells, validating findings from a separate cohort of patients with invasive bladder cancer.^6^

Although CD56 is expressed in many cell types, including tumors, CD56 is often used to designate NK cells in human tumors.^16^, ^17^, ^18^ Therefore, we first examined the prognostic significance of *CD56* gene expression in bladder cancer. We examined the association of *CD56* expression with overall survival (OS) among patients with invasive bladder cancer within TCGA. Unexpectedly, high bladder tumor *CD56* expression was associated with worse OS compared to low bladder tumor *CD56* expression (Figure S1A). Molecular subtypes of bladder cancer have distinct clinical behaviors.^19^ Unsupervised hierarchical clustering with complete linkage revealed two distinct clusters of patients designated as luminal and nonluminal subtypes.^20^ We found that bladder tumors with nonluminal and luminal phenotypes shared biomarkers with nonluminal and luminal breast cancers.^20^ Compared to luminal bladder cancer, nonluminal bladder tumors exhibit increased squamous differentiation, higher rates of metastasis, and shorter patient survival.^21^, ^22^ Bladder tumor *CD56* expression was significantly higher in nonluminal compared to luminal TCGA tumors (Figure S1B). The function of CD56 is not known and many cell types, including tumor cells, can express CD56.^23^ We examined the expression of CD56 by flow cytometric analysis; we found that CD56 is highly expressed on T cells, B cells, myeloid cells, and, to a limited extent, on bladder tumor cells (Figure S2). Collectively, these findings indicate that CD56 by itself is an inadequate marker of functional CD56^bright^ NK cells in bladder tumors and is associated with nonluminal bladder tumor subtype and poor survival outcomes.

**FIGURE 2:**
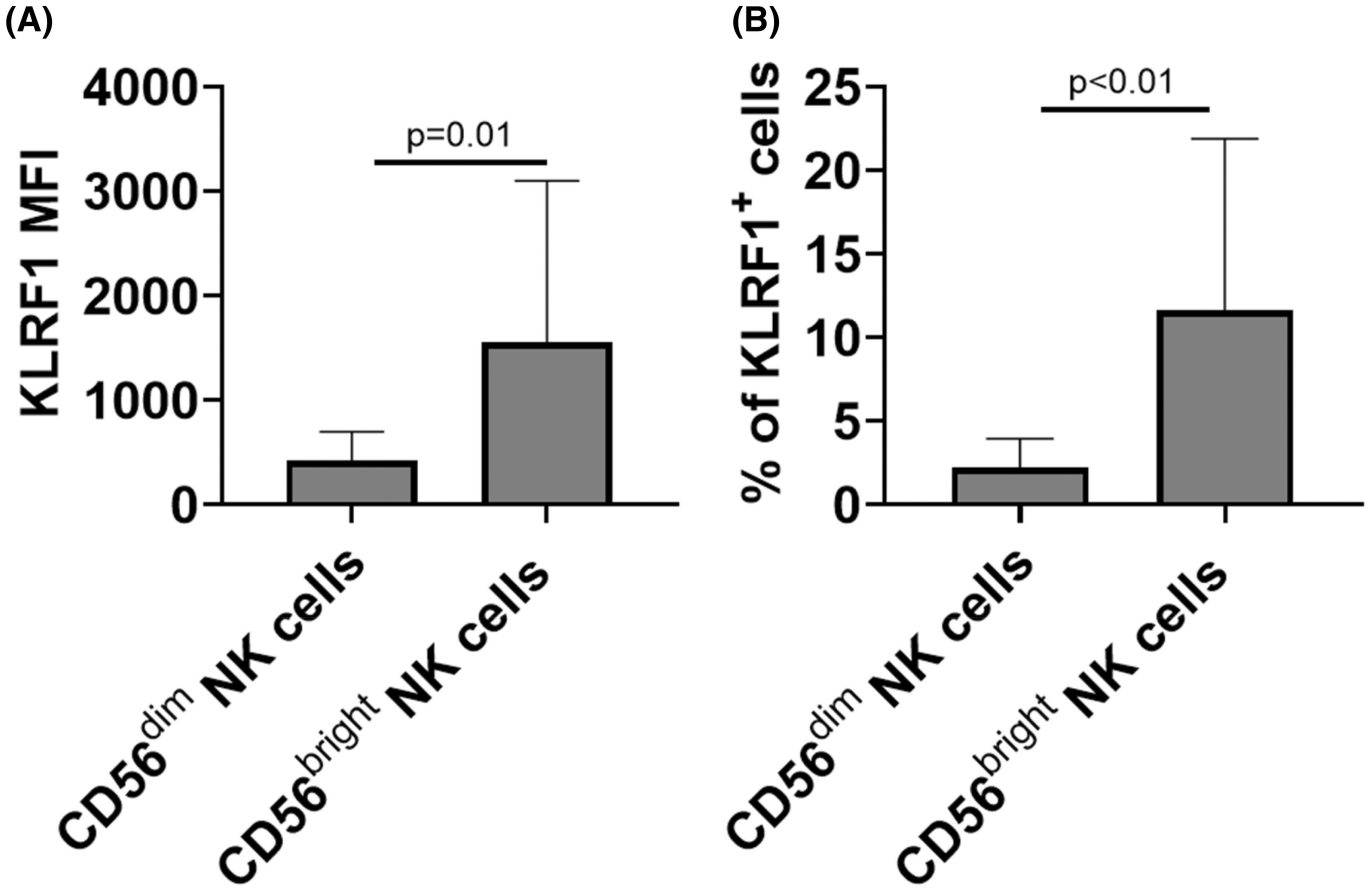
Expression and frequency of KLRF1 are significantly higher in CD56^bright^ NK cells compared with CD56^dim^ NK cells. Human bladder tumor tissues (*n* = 20) were harvested and processed into single‐cell suspensions and analyzed with flow cytometry. KLRF1 MFI (A) and frequency of KLRF1 expression (B) in CD56^bright^ and CD56^dim^ NK cells are plotted. Two‐tailed Mann–Whitney tests.

**FIGURE 3:**
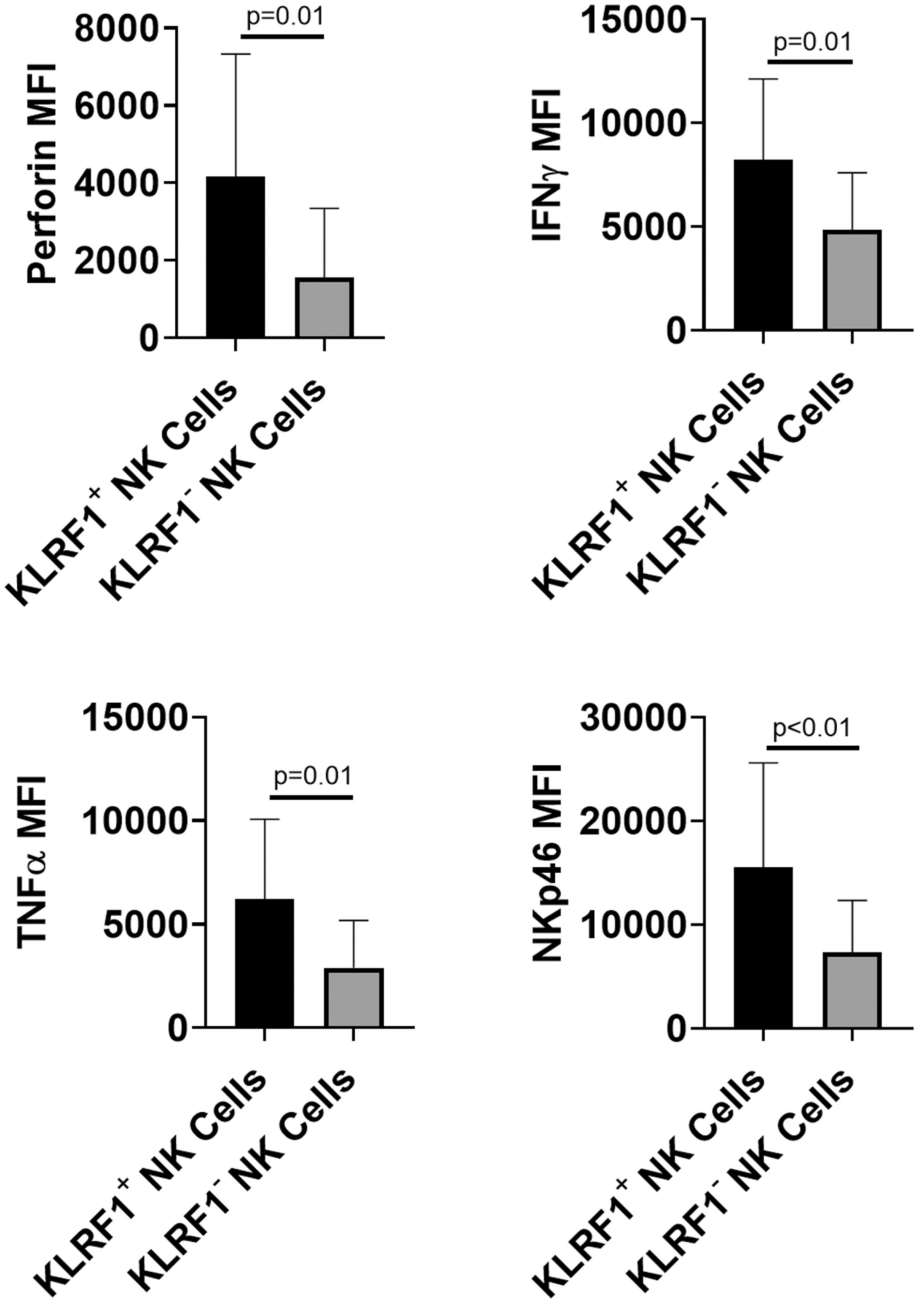
Bladder tumor‐infiltrating KLRF1^+^ NK cells have significantly higher expression of cytokines and activation receptors compared with KLRF1^−^ NK cells. Human bladder tumor tissues (*n* = 20) were harvested and processed into single‐cell suspensions and analyzed with flow cytometry. Different expressions of perforin, cytokines IFNγ and TNFα, and activation receptor (NKp46) in KLRF1^+^ and KLRF1^−^ NK cells are plotted. Two‐tailed Mann–Whitney tests.

A published pan‐cancer analysis of tumors within TCGA identified NK cells by the expression of *KLRF1* and *KLRFC1*.^13^ Examination of invasive bladder tumors in TCGA (*n* = 408, initial bladder cancer patient cohort) showed that *KLRC1* was either absent or expressed at very low levels in most bladder tumors (Figure S3). As a result, there was no difference in the frequency of NK cells between using either *KLRF1* alone or in combination with *KLRC1* in bladder tumor datasets. To determine if KLRF1 surface expression distinguishes CD56^bright^ from CD56^dim^ intratumoral NK cells, we examined KLRF1 expression in CD56^bright^ versus CD56^dim^ NK cells using cohort of patients with invasive bladder cancer (*n* = 20, see Table S4 for clinicpathologic characteristics) and found that KLRF1 expression, as measured by MFI, was significantly increased in CD56^bright^ NK cells compared with CD56^dim^ NK cells (Figure 2A). In addition, the percentage of cells expressing KLRF1 was significantly increased in CD56^bright^ compared to CD56^dim^ NK cells (Figure 2B). KLRF1^+^ NK cells had higher levels of activation markers commonly characterized in functional NK cells including IFNγ (*p* = 0.01), TNFα (*p* = 0.01), perforin (*p* = 0.01), and NKp46 activating receptor (*p* < 0.01) compared with KLRF1^−^ NK cells (Figure 3). These findings support KLRF1 as a surrogate biomarker for the identification of CD56^bright^ NK cells in bladder tumors.

**FIGURE 4:**
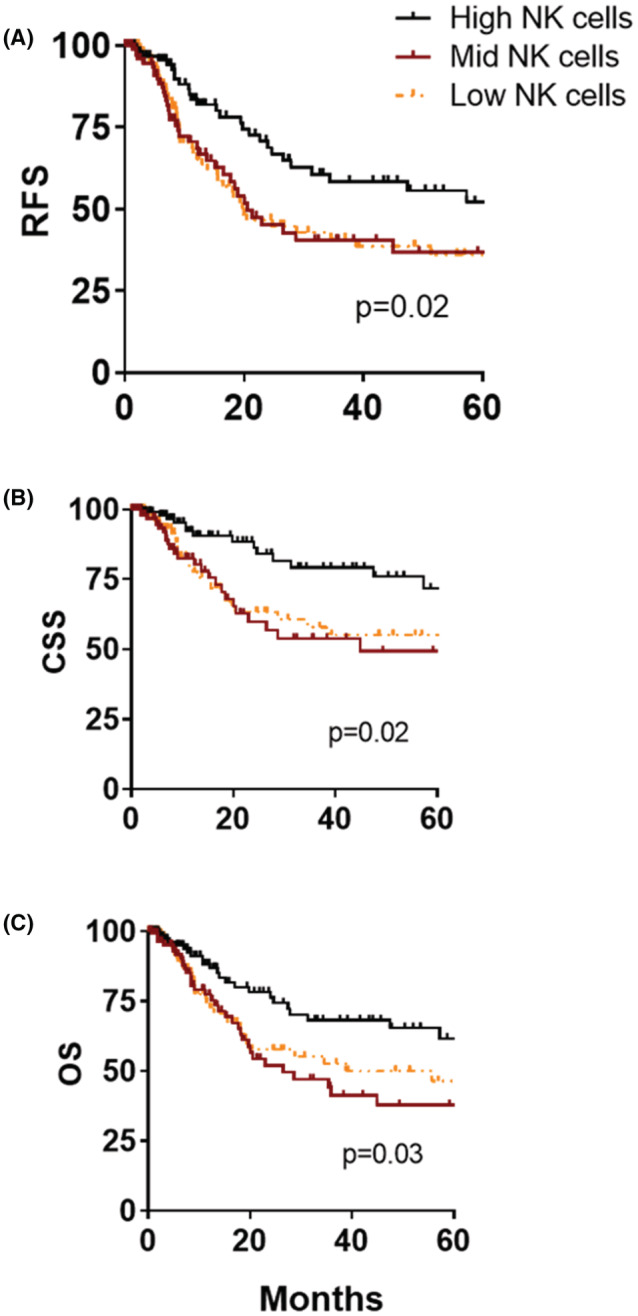
Expression of NK cell marker, *KLRF1*, correlates with improved survival in human bladder cancer. Inventory of TCGA bladder tumor samples (*n* = 351) with cell‐type gene expression markers was based on analysis of Fantom5 data and obtained from online accessible data. Kaplan–Meier plots of recurrence‐free survival (RFS) (A), cancer‐specific survival (CSS) (B), and overall survival (OS) (C), according to tertiles of intratumoral *KLRF1* expression. *p*‐values represent the log‐rank (Mantel‐Cox) test between high and low *KLRF1*.

We next sought to examine the prognostic significance of bladder tumor KLRF1. TCGA analysis revealed that patients with high *KLRF1* bladder tumor expression had significantly improved RFS (*p* = 0.02), CSS (*p* = 0.02), and OS (*p* = 0.03) compared to patients with tumors expressing low levels of KLRF1 (Figure 4, Table 2). The prognostic effect was observed for patients with the highest *KLRF1* expression, whereas no significant difference was observed for patients with mid versus low levels of *KLRF1* (Figure 4). High *KLRF1* expression was also significantly associated with improved RFS (HR = 0.53, *p* = 0.01), CSS (HR = 0.47, *p* = 0.02), and OS (HR = 0.54, *p* = 0.02) on multivariable analysis that included patient age, gender, pathologic stage, tumor *MHC I*, and tumor molecular subtype (Table 3). MHC I is a ligand for inhibitory receptors expressed by NK cells^24^ and the absence of MHC I facilitates the activation of NK cells. Further, we validated our observations in another cohort of *97 high‐grade bladder tumors* ^14^ (downloaded from CBioPortal^15^) and found that KLRF1 is associated with favorable DFS: patients with high *KLRF1* bladder tumor expression had significantly improved DFS (*p* = 0.04, Figure S4), compared to patients with tumors expressing low levels of KLRF1. OS showed a similar trend but did not reach significance (*p* = 0.09).

**TABLE 2:** Univariable analysis for predictors of recurrence‐free survival (RFS), cancer‐specific survival (CSS), and overall survival (OS). Transcription levels as RPKM (reads per kilobase of transcript per million mapped reads) of subtype signature genes and associated clinical data were downloaded from TCGA data portal (*n* = 351 bladder cancer patients). Univariable analysis for predictors of RFS, OS, and CSS was carried out

	RFS		CSS		OS	
	HR (95% CI)	*p*	HR (95% CI)	*p*	HR (95% CI)	*p*
Age	1.02 (1.00–1.04)	0.05	1.03 (0.99–1.05)	0.06	1.04 (1.01–1.06)	<0.01
Gender						
Female	Ref.	–	–	–	–	–
Male	1.07 (0.70–1.64)	0.75	0.84 (0.50–1.41)	0.51	0.83 (0.54–1.27)	0.38
Pathologic stage	1.74 (1.32–2.29)	<0.01	1.94 (1.36–2.77)	<0.01	1.97 (1.46–2.65)	<0.01
Subtype						
Nonluminal	Ref.	–	–	–	–	–
Luminal	0.82 (0.56–1.20)	0.30	0.75 (0.46–1.22)	0.24	0.66 (0.44–0.99)	0.05
KLRF1						
Low	Ref.	–	–	–	–	–
Mid	0.93 (0.60–1.44)	0.75	1.04 (0.60–1.80)	0.90	1.05 (0.66–1.68)	0.82
High	0.54 (0.34–0.87)	0.01	0.48 (0.26–0.90)	0.02	0.57 (0.34–0.95)	0.03
MHC I	0.83 (0.72–0.97)	0.02	0.70 (0.56–0.88)	<0.01	0.78 (0.66–0.92)	<0.01

**TABLE 3:** Multivariable analysis for predictors of recurrence‐free survival (RFS), cancer‐specific survival (CSS), and overall survival (OS). Transcription levels as RPKM (reads per kilobase of transcript per million mapped reads) of subtype signature genes and associated clinical data were downloaded from TCGA data portal (*n* = 351 bladder cancer patients). Multivariable analysis for predictors of RFS, OS, and CSS was carried out

	RFS		CSS		OS	
	HR (95% CI)	*p*	HR (95% CI)	*p*	HR (95% CI)	*p*
Age	1.02 (1.00–1.04)	0.04	1.02 (0.99–1.05)	0.08	1.04 (1.01–1.06)	<0.01
Gender						
Female	Ref.	–	–	–	–	–
Male	0.87 (0.56–1.35)	0.54	0.63 (0.37–1.07)	0.09	0.65 (0.42–1.01)	0.06
Pathologic stage	1.75 (1.31–2.32)	<0.01	1.99 (1.37–2.91)	<0.01	2.07 (1.51–2.83)	<0.01
Subtype						
Nonluminal	Ref.	–	–	–	–	–
Luminal	0.65 (0.43–0.99)	0.04	0.49 (0.29–0.83)	<0.01	0.47 (0.30–0.73)	<0.01
KLRF1						
Low	Ref.	–	–	–	–	–
Mid	0.96 (0.62–1.49)	0.86	1.10 (0.63–1.91)	0.74	1.11 (0.69–1.78)	0.66
High	0.53 (0.33–0.86)	0.01	0.47 (0.25–0.89)	0.02	0.54 (0.32–0.91)	0.02
MHC I	0.83 (0.71–0.98)	0.03	0.67 (0.52–0.86)	<0.01	0.74 (0.61–0.90)	<0.01

Higher KLRF1 expression was observed in tumors expressing high *MHC I* (*p* < 0.01, Table S3). However, the magnitude of the association of *KLRF1* with survival was not modified by tumor *MHC I* as the interaction term *KLRF1‐MHC I* was not significant in multivariable models of RFS, CSS, and OS (*p* ≥ 0.70). The association of *KLRF1* with favorable survival outcomes remained after adjustment for adjuvant therapy, including radiation and chemotherapy (*p* ≤ 0.04 for RFS, CSS, and OS; *n* = 221 patients; Table S4).

## 4 DISCUSSION

In this study, examination of CD56^bright^ NK cells revealed a more activated phenotype compared to CD56^dim^ NK cells, including increased expression of activation markers, perforin, and IFNγ. The activation marker, KLRF1, was abundantly expressed on CD56^bright^ NK cells but poorly on CD56^dim^ NK cells. KLRF1^+^ NK cells had higher levels of activation markers commonly characterized in functional NK cells. Finally, intratumoral KLRF1was identified as a novel biomarker in bladder cancer as it was associated with favorable survival outcomes in patients undergoing cystectomy.

NK cells are critical members of the innate immune system which mediate antitumor responses through their cytolytic and cytokine‐producing abilities.^25^ Evidence for the prognostic significance of NK cells in solid tumors is emerging. In 2020, Zhang et al.^26^ carried out a meta‐analysis that investigated the prognostic significance of tumor‐infiltrating NK cells in solid tumors. They found that high levels of NK cell markers (CD56, CD57, NKp30, and NKp46) in solid tumor tissues correlated with improved overall survival in cancer patients. In contrast, our findings show that in bladder cancer, *CD56* expression alone was not associated with improved survival and, in fact, was associated with nonluminal subtypes and worse outcomes. CD56 was inadequate in identifying activated NK cells in bladder cancer. This discrepancy in findings could be due to the large number of non‐NK cells that express CD56 in bladder tumors, making CD56 a poor marker for functional NK cells in bladder tumors.

The functional significance of KLRF1 in bladder cancer NK cells is unknown. KLRF1 was initially identified as a part of human cDNA which displayed homology to human NKRP1A from an expressed sequence tag database.^27^ Activation of KLRF1 receptor on NK cells mediates calcium mobilization and cytotoxic effects.^28^ KLRF1 was also identified as a marker of NK cell maturity in secondary lymphoid tissues.^29^ Thus, high *KLRF1* gene expression potentially marks more mature and activated NK cells in bladder tumors, supported by the strong association of high *KLRF1* with improved survival.

A higher frequency of intratumoral *KLRF1*‐expressing NK cells was observed in tumors with high *MHC I* expression. Given the role of MHC I in diminishing NK cell function, this finding was surprising. Expression of MHC I by tumors is directed by the interaction of tumors with immune cells in the microenvironment. In preclinical models, induction of MHC I by tumors occurs concomitantly with infiltration of the tumor by immune cells, and NK cells, in particular, regulate MHC I expression through the production of IFN‐γ.^30^ The significant association of intratumoral NK cells with tumor *MHC I* expression observed in this cohort could be explained by NK cell modification of tumor immunogenic profile through MHC I upregulation as proposed.^30^ In this regard, NK cells upregulation of bladder tumor MHC I could then facilitate T‐cell‐mediated killing.

In addition to their role in upregulating tumor MHC I and activating cytotoxic T cells, NK cells also mediate direct tumor cytotoxicity. MHC I is an important inhibitory ligand that binds to and diminishes the effector function of NK cells.^31^ Because tumor MHC I decreases NK cell cytotoxic function, we predicted that intratumoral NK cells would be most prognostic in tumors expressing low MHC I. However, the prognostic ability of intratumoral NK cells was not significantly modified by tumor MHC I expression. It is possible that the magnitude of influence of tumor MHC I on the association of NK cells with survival was too small to be detected with our sample size. Alternatively, intratumoral NK cells could mediate protective antitumor mechanisms even in high MHC I tumors which could account for their prognostic relevance in high MHC I tumors. These findings also suggest that NK cell‐directed therapies could be effective in both low and high MHC I‐expressing tumors.

This study has important limitations. Conclusions drawn from the TCGA dataset are limited by their inability to map genetic and protein differences to the single cells or distinct cellular populations within the tumor. The identification strategy for NK cells is subject to criticism because the markers used to identify NK cells are diverse and utilizing KLRF1 marks more activated NK cell phenotypes. Although CD56^dim^ NK cells expressed lower levels of KLRF1 compared to CD56^bright^ NK cells, the expression of KLRF1 within CD56^bright^ NK cells was variable (shown by wide error bars in Figure 2A). In addition, the RFS K‐M estimates were similar for patients with low versus mid KLRF1‐expressing tumors. Therefore, reproducibility of biomarker performance based on a precise and relatively high KLRF‐1 expression cut‐point is required to validate these findings. Further, we were unable to explore inactive NK cell phenotypes including the CD56^dim^ population that we previously reported as being dysfunctional.^6^ There are drawbacks to the categorization of NK cells into quantiles, including multiple testing, homogeneity within risk groups, and the ability to compare to other tests. However, grouping NK cells into tertiles revealed an important lack of prognostic distinction between patients expressing low and mid‐levels of intratumoral NK cells. Further, the number of samples used to perform flow and mass cytometry analysis is limited, but the findings were validated in multiple patient cohorts. Another limitation is the time between tissue acquisitions from patients undergoing surgery at the clinical site and tissue processing in the laboratory which may result in lower cell yield and influence the content and functions of immune cells but extreme precautions (such as specialized tissue collection media and controlled temperature during tissue transport) are taken to reduce such variations. Lastly, the use of KLRF1 in identifying NK cells is controversial because a uniformly agreed‐upon standard strategy for identifying activated NK cells does not exist. However, our strategy of using KLRF1 as an identification strategy for activated NK cells is based on previously published studies. Expression of other NK cell markers such as KLRC3, KLRD1, and NCR1, although useful, were not investigated to maintain focus. A comparative analysis between identification strategies of NK cells using different markers should be investigated in future studies and will generate important insight into the phenotypes of NK cells in bladder cancer. Despite these limitations, this study validates the prognostic importance of NK cells in bladder cancer and makes several novel observations that may impact the development of new therapies.

## 5 CONCLUSIONS

Bladder CD56 expression is associated with more aggressive nonluminal tumors and poor survival outcomes in bladder cancer. In contrast, KLRF1 is a marker of bladder tumor‐infiltrating‐activated CD56^bright^ NK cells and is associated with improved survival for patients with invasive bladder cancer. These data support KLRF1 as a potential prognostic marker in bladder cancer but this prognostic significance requires validation.

## AUTHOR CONTRIBUTIONS

**Neelam Mukherjee:** Conceptualization (lead); data curation (lead); formal analysis (lead); writing – original draft (lead). **Niannian Ji:** Data curation (supporting); formal analysis (supporting). **Xi Tan:** Data curation (supporting); formal analysis (supporting). **Chun‐Liang Chen:** Formal analysis (supporting); methodology (supporting). **Onika DV Noel:** Data curation (equal); formal analysis (equal). **Maria Rodriguez‐Padron:** Writing – review and editing (supporting). **Chun‐Lin Lin:** Formal analysis (supporting). **David G. Alonzo:** Writing – review and editing (supporting). **Tim H.‐M. Huang:** Conceptualization (supporting); resources (supporting); software (supporting); supervision (supporting). **Robert Svatek:** Conceptualization (equal); funding acquisition (lead); resources (lead); supervision (lead); writing – original draft (supporting).

## FUNDING INFORMATION

The Glenda and Gary Woods President's Distinguished Chair in GU Oncology. The Roger L. and Laura D. Zeller Charitable Foundation Chair in Urologic Cancer. Ron and Karen Herrmann Department of Urology Education Endowment. Max and Minnie Tomerlin Voelcker Fund. NIH 5K23CA178204‐03. Bladder Cancer Advocacy Network (BCAN). CPRIT‐funded Institutional Research Training Award (RTA; RP170345). Mays Cancer Center P30 Cancer Center Support Grant (National Cancer Institute) (CA054174). Long School of Medicine at UTHSCSA and the Institute for the Integration of Medicine and Science.

## CONFLICT OF INTEREST

The authors have declared that no conflict of interest exists.

## Supporting information


Data S1.

## Data Availability

The data that support the findings of this study are available from the corresponding author upon reasonable request.
