# Supplementary material for: KLRF1, a novel marker of CD56bright NK cells, predicts improved survival for patients with locally advanced bladder cancer
Source: Cancer Med. 2022 Dec 29;12(7):8970–80. doi: 10.1002/cam4.5579 (PMC10134305; doi:10.1002/cam4.5579)
Supplement: Supplementary file 1 — Data S1. [file CAM4-12-8970-s001.docx]

**Supplementary Tables**

**Supplementary Table 1. Clones and metal labels of antibodies (CyTOF).** Clones and metal labels of antibodies used in the CyTOF analysis are listed.


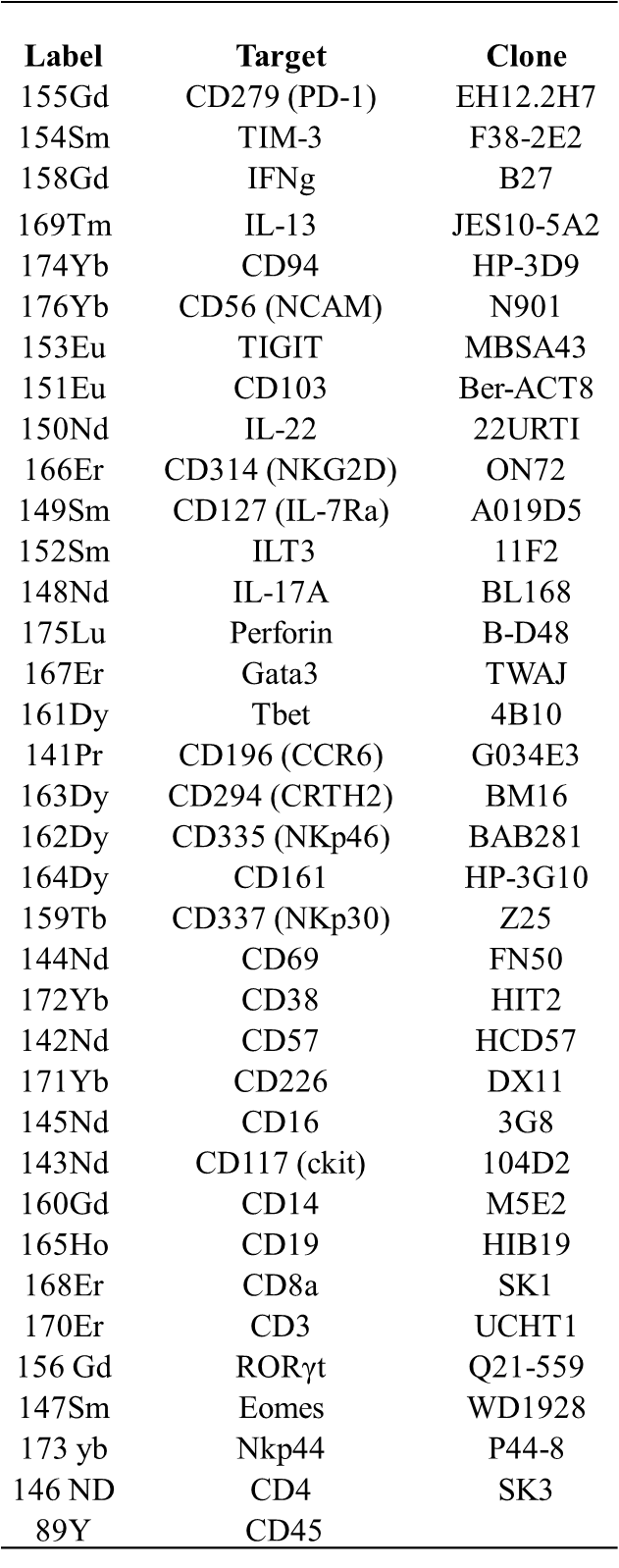


**Supplementary Table 1. Clones and metal labels of antibodies (CyTOF).** Clones and metal labels of antibodies used in the CyTOF analysis are listed.

**Supplementary Table 2. Clones and fluorophores of antibodies (Cytek).** Clones and fluorophores of antibodies used in the Cytek flow analysis are listed.


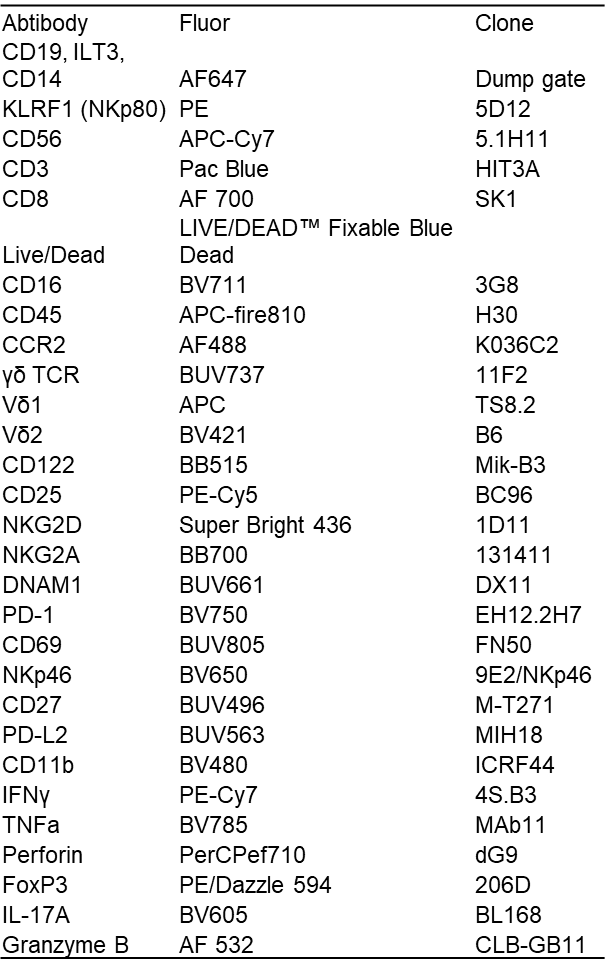


**Supplementary Table 3. Association of KLRF1 with clinical and pathologic variables.** Transcription levels as RPKM (reads per kilobase of transcript per million mapped reads) of subtype signature genes and associated clinical data were downloaded from TCGA data portal (n=351 bladder cancer patients). Association of KLRF1 with clinical and pathologic variables were analyzed.

| **Variable** | **Median KLRF1 frequency [IQR]** | **p-value** |
| --- | --- | --- |
| Gender |  | 0.40 |
| Female | 0.20 [0.08, 0.46] |  |
| Male | 0.17 [0.07, 0.52] |  |
| Pathologic T stage |  | 0.80 |
| ≤pT2 | 0.18 [0.05, 0.43] |  |
| >pT2 | 0.17 [0.09, 0.52] |  |
| Node Positivity |  | 0.69 |
| No | 0.18 [0.07, 0.45] |  |
| Yes | 0.16 [0.08, 0.53] |  |
| Subtype |  | 0.54 |
| Non-luminal | 0.18 [0.08, 0.46] |  |
| Luminal | 0.16 [0.06, 0.56] |  |
| Tumor MHC-1 expression |  | <0.01 |
| High | 0.23 [0.10, 0.60] |  |
| Low | 0.13 [0.05, 0.29] |  |
| Adjuvant Chemotherapy |  | 0.46 |
| No | 0.18 [0.06, 0.57] |  |
| Yes | 0.22 {0.85, 0.66] |  |
| Adjuvant Radiation |  | 0.93 |
| No | 0.19 [0.07, 0.57] |  |
| Yes | 0.16 [0.07, 0.86] |  |

**Supplementary Table 4. Multivariable analysis for predictors of recurrence-free survival**

**(RFS), overall survival (OS) and cancer-specific survival (CSS) with adjuvant therapy.**

Association of RFS, OS and CSS with adjuvant therapy were analyzed in a multivariate model (n=221 patinets).

|  | **RFS** | |  | **CSS** | |  | **OS** | |
| --- | --- | --- | --- | --- | --- | --- | --- | --- |
|  | **HR (95% CI)** | **p** |  | **HR (95% CI)** | **p** |  | **HR (95% CI)** | **p** |
| Age | 1.02 (1.00-1.04) | 0.11 |  | 1.03 (1.00-1.07) | 0.07 |  | 1.04 (1.01-1.07) | 0.02 |
| Gender  Female  Male | Ref.  0.85 (0.51-1.43) | -  0.55 |  | -  0.51 (0.27-0.95) | -  0.04 |  | -  0.61 (0.35-1.04) | -  0.07 |
| Pathologic stage | 1.59 (1.13-2.24) | 0.01 |  | 1.55 (0.98-2.45) | 0.06 |  | 1.69 (1.15-2.50) | 0.01 |
| Subtype  Non-Luminal  Luminal | Ref.  0.71 (0.43-1.19) | -  0.19 |  | -  0.49 (0.26-0.92) | -  0.03 |  | -  0.53 (0.30-0.92) | -  0.02 |
| NK cells  Low  Mid  High | Ref.  1.08 (0.64-1.91)  0.52 (0.30-0.91) | -  0.71  0.02 |  | -  1.10 (0.56-2.18)  0.40 (0.19-0.85) | -  0.78  0.02 |  | -  1.20 (0.66-2.18)  0.51 (0.28-0.96) | -  0.56  0.04 |
| MHC I | 0.92 (0.76-1.12) | 0.40 |  | 0.67 (0.48-0.92) | 0.01 |  | 0.75 (0.59-0.96) | 0.02 |
| Adjuvant chemotherapy  No  Yes | Ref.  0.82 (0.48-1.41) | -  0.48 |  | -  1.44 (0.73-2.82) | -  0.29 |  | -  0.90 (0.49-1.65) | -  0.73 |
| Adjuvant radiation therapy  No  Yes | Ref.  1.02 (0.34-3.06) | -  0.98 |  | -  1.28e-15 (0-.) | -  1.00 |  | -  0.36 (0.05-2.73) | -  0.32 |

**Supplementary Figures**


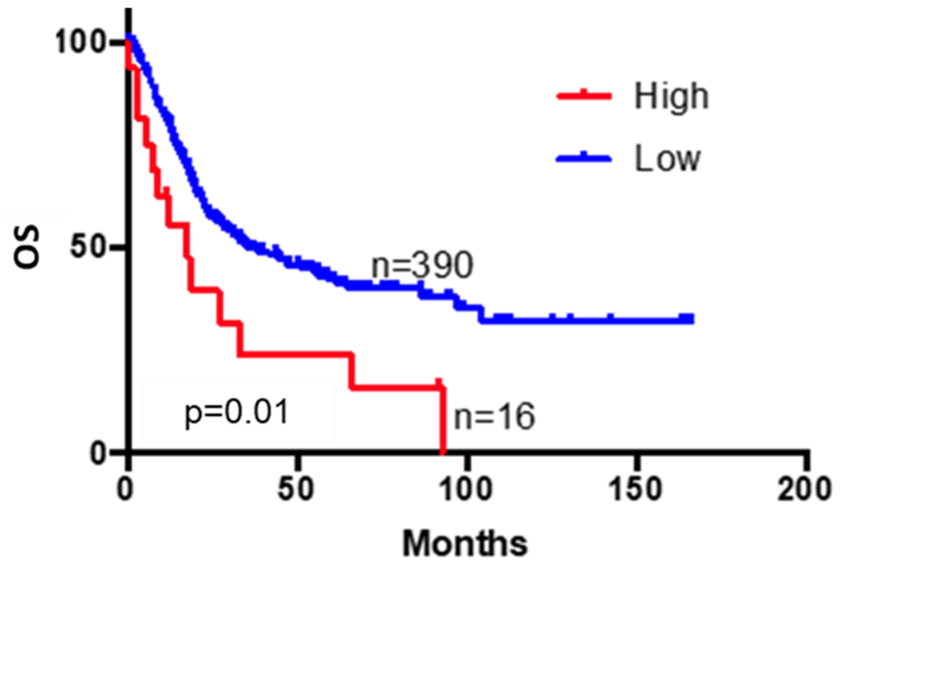


***CD56***

**A**

**B**

***CD56***


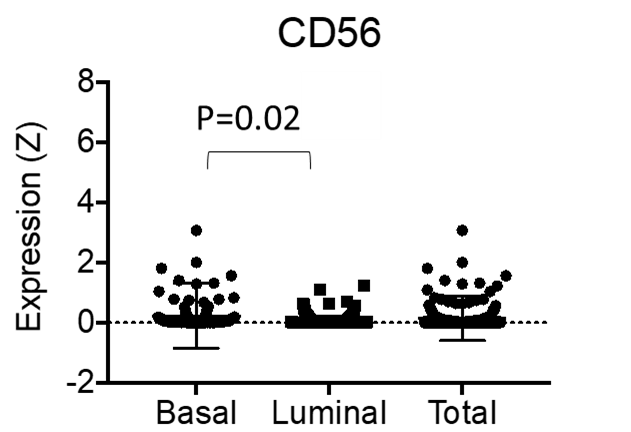


**Supplementary Figure 1. *CD56* expression is associated with worse overall survival and the more aggressive basal tumor subtype** (**A)** The Kaplan-Meyer curve estimation indicates that higher *CD56* expression levels in tumors predict worse overall survival in the TCGA bladder cancer patient cohort. The *CD56* expression is based on mRNA-seq data (z-scores) of TCGA. The patients with top 4% *CD56* expression levels with at least half a standard deviation (0.37) above the mean (0.14) were considered as the high group vs the rest as the low group. p-values represent the Log-rank test. The sizes of patient groups are shown over the K-M curves. (**B**) The expression of CD56 in basal subtype tumors (n=182) is significantly higher than in the luminal subtype (n=226) in the TCGA bladder cancer patient cohort. The patient tumor subtypes were stratified according to Lotan et al 2019 ^20^.


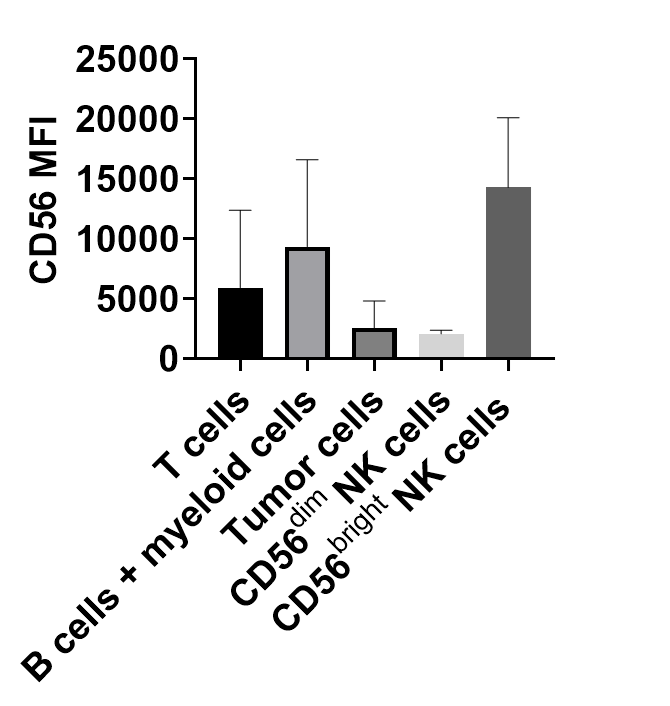


**Supplementary Figure 2. CD56 expression in immune and tumor cells.** Human bladder tumor tissues (n=20) were harvested and processed into single cell suspensions and analyzed with flow cytometry. T cells were identified as CD45^+^CD3^+^ cells, B cells and myeloid cells were identified together as CD45^+^CD19^+,^CD14^+^ILT3^+^ cells, tumor cells as CD45^-^ cells, and NK cells as CD45^+^, CD3^–^, CD14^–^, CD19^–^, ILT3^–^ and CD56^dim/bright^. CD56 MFI in indicated cells is plotted.


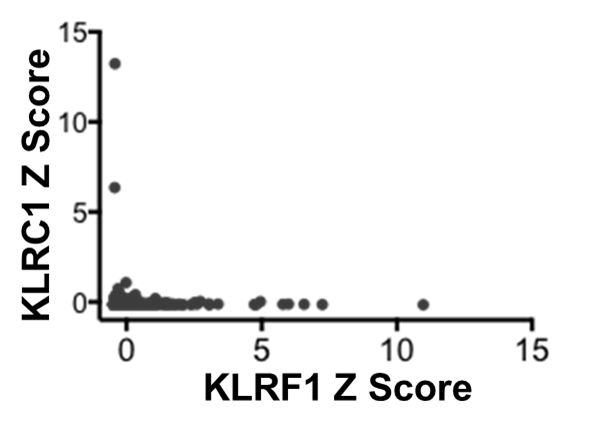


**Supplementary Figure 3. Expression of *KLRC1* is absent or low in most human bladder tumors.** The scatter plot displays the expression of *KLRF1* and *KLRC1* in the tumor samples (n=408) from the TCGA bladder cancer patient cohort including the n=351 patients that were used for the final analysis. The gene expression data were obtained from TCGA RNA-seq of bladder cancer patient cohort samples. The means of KLRF1 and KLRC1 expression are 0.0644 and -0.04503, respectively. Pearson correlation analysis showed the expression of KLRF1 and KLRC1 are not concordant (r= -0.033, p=0.58).

**
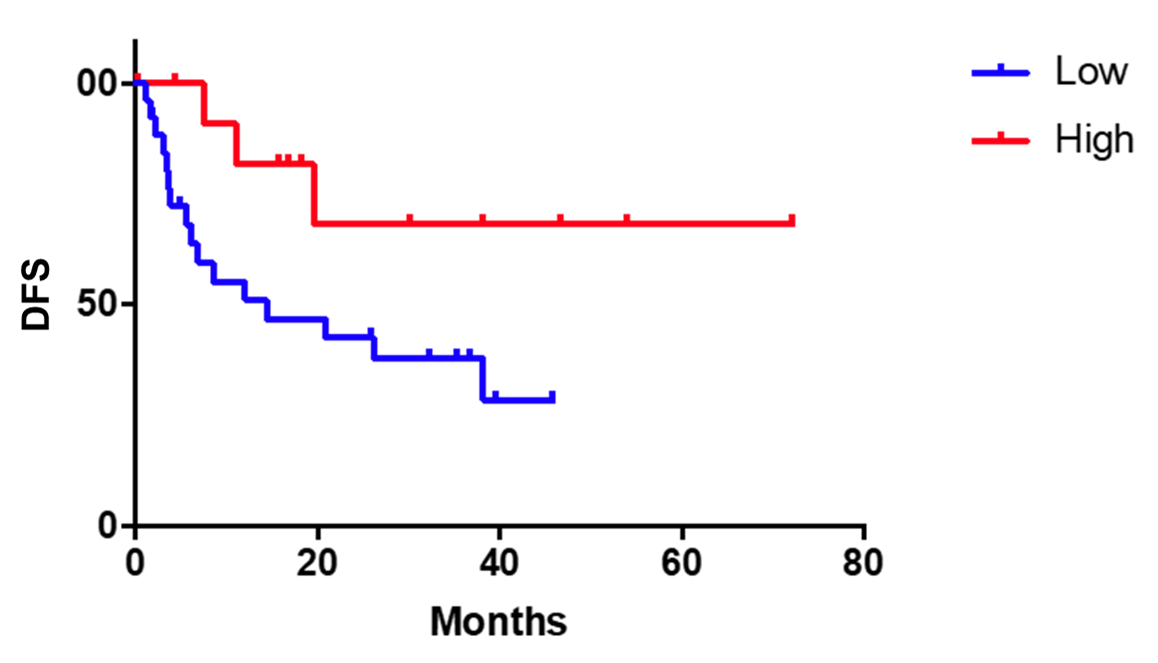
**

p=0.0433

**Supplementary Figure 4. KLRF1 expression correlates with increased disease-free survival (DFS) in high-grade bladder tumors.** The gene expression and clinical data of the patient cohort with high-grade bladder cancer [14] were downloaded from CBioPortal. For Kaplan-Meier estimation analysis, the groups of high and low KLRF1 expression were based on a Z-score value of -0.2.
